# Visuomotor Map Determines How Visually Guided Reaching Movements are Corrected Within and Across Trials^1^,^2^,^3^

**DOI:** 10.1523/ENEURO.0032-16.2016

**Published:** 2016-06-03

**Authors:** Takuji Hayashi, Atsushi Yokoi, Masaya Hirashima, Daichi Nozaki

**Affiliations:** 1 Division of Physical and Health Education, Graduate School of Education, The University of Tokyo, Tokyo 113-0033, Japan; 2 Japan Society for the Promotion of Science, Tokyo 102-8471, Japan; 3 The Brain and Mind Institute, University of Western Ontario, London, Ontario, Canada N6A 5B7; 4 Graduate School of Frontier Biosciences, Osaka University, Suita, Osaka 565-0871, Japan; 5 Center for Information and Neural Networks, National Institute of Information and Communications Technology, Suita, Osaka 565-0871, Japan

**Keywords:** feedback control, feedforward control, motor adaptation, reaching movement, visuomotor map

## Abstract

When a visually guided reaching movement is unexpectedly perturbed, it is implicitly corrected in two ways: immediately after the perturbation by feedback control (online correction) and in the next movement by adjusting feedforward motor commands (offline correction or motor adaptation). Although recent studies have revealed a close relationship between feedback and feedforward controls, the nature of this relationship is not yet fully understood. Here, we show that both implicit online and offline movement corrections utilize the same visuomotor map for feedforward movement control that transforms the spatial location of visual objects into appropriate motor commands. First, we artificially distorted the visuomotor map by applying opposite visual rotations to the cursor representing the hand position while human participants reached for two different targets. This procedure implicitly altered the visuomotor map so that changes in the movement direction to the target location were more insensitive or more sensitive. Then, we examined how such visuomotor map distortion influenced online movement correction by suddenly changing the target location. The magnitude of online movement correction was altered according to the shape of the visuomotor map. We also examined offline movement correction; the aftereffect induced by visual rotation in the previous trial was modulated according to the shape of the visuomotor map. These results highlighted the importance of the visuomotor map as a foundation for implicit motor control mechanisms and the intimate relationship between feedforward control, feedback control, and motor adaptation.

## Significance Statement

Feedforward control of reaching movements relies on a visuomotor map that translates motor planning, based on the target’s location, into an appropriate movement. However, movements could be unexpectedly perturbed, indicating that additional mechanisms for movement corrections are necessary. We hypothesize that the visuomotor map provides the motor system with the knowledge of how the movement should be corrected during the movement (feedback control) and in the next movement (motor adaptation). We demonstrate that distorting the visuomotor map, by a visuomotor adaptation paradigm, alters the magnitudes of both movement corrections according to the shape of the distortion. Our results show the significance of visuomotor map and intimate link between feedforward control, feedback control, and motor adaptation through a visuomotor map.

## Introduction

**Figure 1.**
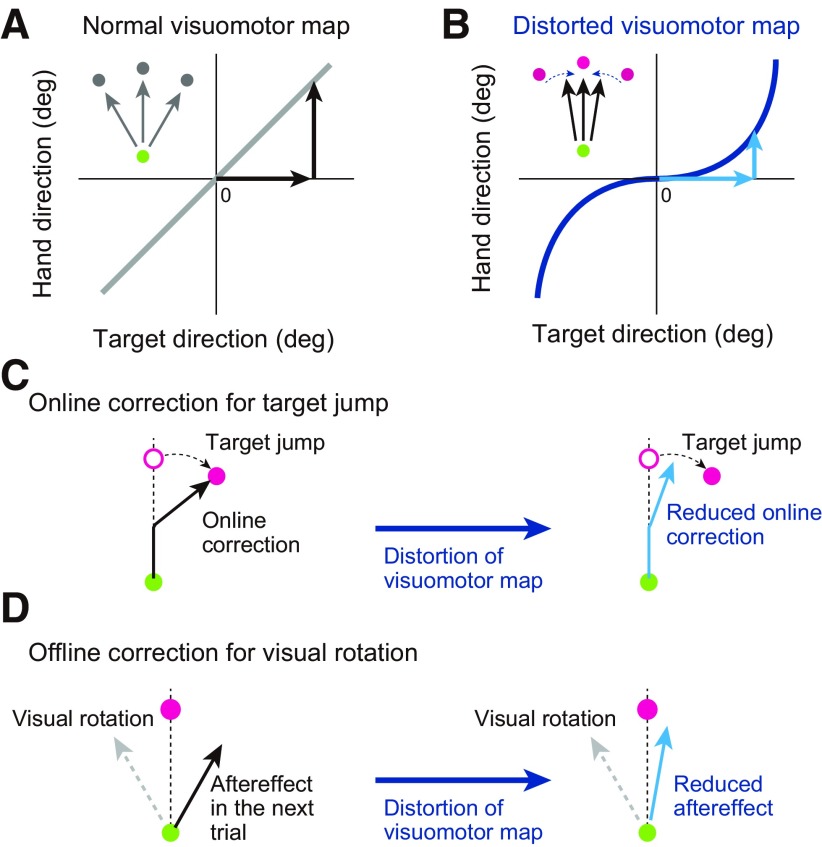
Schematic representation of our hypothesis. The visuomotor map is the relationship between the target direction and hand movement direction. ***A***, In ordinary situations, these two directions are almost identical. ***B***, If the map can be distorted (represented by a blue solid line), a voluntary reaching movement toward three targets should always result in the same hand movement directed to 0°. Participants cannot voluntarily change the movement direction even if they try. ***C***, ***D***, We hypothesized that online movement correction to a target jump (***C***) and offline movement correction observed after imposing visual rotation (***D***) also cannot be changed appropriately.

Our ability to accurately reach toward visual objects is achieved by feedforward control based on a visuomotor map that transforms the spatial information of a target location into an appropriate movement (Fig. 1*A*) (Johnson et al., 1996; Kalaska et al., 1997; Wise et al., 1997; Pouget and Snyder, 2000). However, feedforward control is not always versatile; the movement could be perturbed by noise and/or uncertainty within our nervous system and environments (Harris and Wolpert, 1998; Hamilton et al., 2004; Faisal et al., 2008; Franklin and Wolpert, 2011). Facing such unexpected perturbations, the motor system corrects movements in two different ways. Consider a laboratory situation in which a participant is making a reaching movement toward a visual target. When a cursor representing their hand deviates from the target, the motor system can correct the movement during the movement by feedback control (online correction; Diedrichsen, 2007, 2010; Scott et al., 2015) and in the next trial by motor adaptation (offline correction; Shadmehr and Mussa-Ivaldi, 1994; Shadmehr et al., 2010; Wolpert et al., 2011).

Intriguingly, these movement corrections are not necessarily achieved voluntarily (Goodale et al., 1986; Kagerer et al., 1997; Desmurget et al., 1999; Kasuga et al., 2013). Online correction during movements begins very rapidly (<150 ms) after visual perturbation (Franklin and Wolpert, 2008; Dimitriou et al., 2013; Reichenbach et al., 2014), much faster than voluntary movement correction (Day and Lyon, 2000; Franklin and Wolpert, 2008; Kobak and Mehring, 2012). As for offline correction, in the trial immediately after the perturbation, the movement inevitably deviates in the opposite direction (i.e., aftereffect), even if participants aim at the target (Wei and Körding, 2009; Kasuga et al., 2013).

Here, we hypothesized that the visuomotor map for voluntary movement control could influence the implicit online and offline movement corrections. To test the causal links between them, we examined how these movement corrections were influenced when the visuomotor map was artificially distorted. Theoretically, if we can distort the visuomotor map as shown in Figure 1*B*, this should result in reaching movements that are less sensitive to differences in the target’s direction. Therefore, if both movement corrections refer to the visuomotor map, both types of movement corrections should also decrease following distortion of the visuomotor map. On the other hand, if the movement corrections are independent of the visuomotor map, we should observe the same amount of movement correction, even after the shape of the visuomotor map has been distorted. We tested this prediction by examining the rapid online correction when the target location was moved to another place immediately after the initiation of movement (Fig. 1*C*) and the offline correction in the trial immediately after the deviation was imposed on the cursor (Fig. 1*D*).

## Materials and Methods

### Participants

Fifty-four right-handed participants (34 males and 20 females; age 15–52 years old) with no reported neurological disorders participated after giving informed consent. The entire protocols were approved by the ethics committee of our university.

### General task settings

The participants performed reaching movements with their right arm in a horizontal plane while holding the handle of a robotic manipulandum (KINARM End-point Lab, BKIN Technologies). Their trunks were fixed to the chair by two belts at right and left shoulders. Wrist movements were constrained by a brace, and an arm sling was used to support the upper arm horizontally and maintain a constant posture. A white cursor representing the handle position (10 mm diameter), a starting circle (14 mm diameter), and a target circle (14 mm diameter) were presented via a mirror placed over the arm, which occluded direct vision of participants’ own arm. They were instructed to move the cursor from the starting circle toward the target circle (movement distance: 15 cm). A warning message “fast” or “slow” was displayed just below the start position if the movement velocity was outside 600–750 mm/s (Experiments 1 and 3) and 380–450 mm/s (Experiment 2). In Experiment 2, slow movement was used, so that participants had sufficient time for online movement correction.

Before each trial, the participants moved the cursor into the green start circle. After 1 s holding period, the green circle appeared at target positions. After 1–1.5 s, the target’s color turned magenta, indicating “go”. Participants were required to move the cursor as accurately as possible toward the target circle. After the completion of each trial, the KINARM robot automatically returned the handle to the start position. Participants were asked not to return the handle by themselves.

### Procedure for distortion of the visuomotor map

We defined the visuomotor map as the relationship between the target direction and actual hand movement direction. In ordinary situations, these two directions should be almost identical, because we can move our hands accurately to targets located anywhere. We attempted to distort this visuomotor map. In Experiment 1, we confirmed the visuomotor map was altered. Experiments 2 and 3 examined how online (Experiment 2) and offline (Experiment 3) movement corrections were influenced by distortion of the visuomotor map.

**Figure 2.**
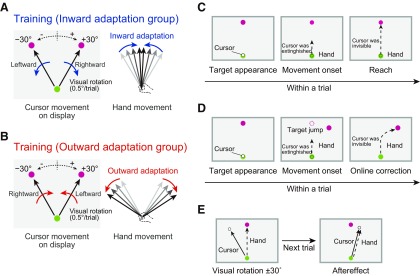
Experimental setup. Participants alternately reached toward one of two targets located rightward and leftward. ***A***, In inward adaptation group, gradually increasing rightward and leftward visual rotations were imposed on the cursor when reaching to rightward and leftward targets, respectively. This procedure would make the handle movements closer to the target. Participants were not aware of the presence of visual rotation. ***B***, In the outward adaptation group, the association of target and visual rotation was reversed, making the handle movements more distant. ***C***–***E***, the participants performed probe trials in order to obtain the visuomotor map (***C***), online (***D***), and offline movement correction (***E***) in Experiments 1, 2, and 3, respectively (see Materials and Methods).

To distort the visuomotor map, we used the following method. The target was displayed alternately 30° to either the right or left of the straight-ahead position (0°). When reaching to the right or left target, rightward or leftward visual rotation around the starting position was applied to the cursor representing the handle position (Fig. 2*A*). The amount of visual rotation was increased gradually from 0° to 30°, at a rate of 0.5° a trial (61 trials for each target) so participants were not aware of the presence of visual rotation. This procedure implicitly made the movement direction of the handle closer (i.e., inward) even when the participants aimed at the two different targets (Hirashima and Nozaki, 2012). We called the participants who experienced this training the inward adaptation group.

We also used another type of intervention, in which visual rotations were applied in the opposite direction (Fig. 2*B*). Specifically, when reaching to the right or left targets, leftward or rightward visual rotation around the starting position was applied to the cursor representing the handle position. These rotations made the movement direction of the hand become more distant (i.e., outward). We called participants who experienced this training the outward adaptation group. If participants noticed the presence of visual rotations, the experiment was terminated and the data abolished. In total, the data of four participants (2 each for Experiments 2 and 3) were discarded. In the following sections, the data did not contain these participants.

### Experiment 1: distortion of visuomotor map

To confirm the visuomotor map was actually distorted by the intervention described above, we obtained the visuomotor map by having participants (*N* = 6 for each of inward and outward adaptation group) reach to targets located at various positions (0°, ±7.5°, ±15°, ±30°, ±45°, and ±60°; 6 trials for each target) without visual feedback (i.e., the cursor was invisible) before and after the intervention. Before the intervention, participants were asked to reach toward each target (Fig. 2*C*), and the cursor became invisible immediately after the color of the target changed. After the intervention, participants reached alternately to 2 targets located at ±30˚ under 30˚ visual rotation. Probe trials to each target were randomly interleaved (66 probe trials out of 266 total trials). In the probe trials, the cursor became invisible immediately after the color of the target changed so that unnecessary visuomotor adaptation did not occur. After adapting to the inward (or outward) adaptation, we expected that the movement direction would become less sensitive (or more sensitive) to changes in the target locations.

### Experiment 2: alteration of feedback control

Sixteen participants were assigned to two groups (*N* = 8 for each group) according to the type of distortion of visuomotor map (inward or outward adaptation; see Experiment 1). To investigate how the intervention influenced online movement correction, we adopted an experimental paradigm using target jump (Fig. 2*D*; Goodale et al., 1986; Desmurget et al., 1999; Day and Lyon, 2000; Izawa and Shadmehr, 2008; Gritsenko et al., 2009; Gritsenko and Kalaska, 2010). As shown in Experiment 1, movement to the central target remained unchanged after intervention. Thus, we used movement to the central target as probe trials to investigate online movement correction; more specifically, in probe trials reaching toward the central target, the target location changed to another location (±30°, ±15°, ±7.5°, 0°) after the force to the handle exceeded 1 N when starting reaching movement. The cursor disappeared simultaneously so that unnecessary adaptation did not occur during online movement correction.

Participants performed 100 reaching movements toward each target located at ±30°, in alternate fashion. A reaching movement to the central target was randomly interleaved (100 trials). Thirty trials were performed with visual feedback (i.e., the cursor was visible), and the remaining 70 trials were probe trials without visual feedback (i.e., target jump trials; 10 trials for each size of target jump). These procedures were performed before and after intervention.

### Experiment 3: alteration of aftereffect

We investigated how intervention influenced offline movement corrections. Twenty-two participants were randomly assigned to two groups (*N* = 11 for each group) according to the type of visuomotor map distortion (inward or outward adaptation; see Experiment 1). As in Experiment 2, we used movement to the central target as probe trials. Visual rotations (−30°, 0°, 30°) were applied to the cursor when participants reached to the central target. In the next trial to reach the same central target, we measured the corrected movement direction in the direction opposite the visual rotation (i.e., aftereffect; Fig. 2*E*). We asked the participants to aim at the central target as accurately as possible and not use explicit strategy (Taylor et al., 2014) to change the movement direction. In perturbed trials, we also asked participants not to correct during the movement, so that online movement correction did not influence offline movement correction.

Participants performed 120 reaching movements toward each target located at ±30° positions in alternate fashion. A pair of one visual rotation trial and one probe trial was randomly interleaved (10 pairs for each of 0°, +30°, and −30° visual rotations). These procedures were performed before and after intervention.

### Data analysis

Data on the kinematics of the handle (position and velocity) and the force on the handle were sampled at 1000 Hz and filtered with a cutoff frequency of 10 Hz using a fourth-ordered Butterworth filter. For the analysis of Experiment 1, we obtained the movement direction by calculating the angle of the line connecting the starting position and handle position at the peak velocity relative to the forward direction. The rightward and leftward movements were defined as positive and negative, respectively.

In the probe trials for Experiment 2, the visual cursor was turned off. To ensure if online movement corrections were performed appropriately even when visual feedback was not available, we calculated the angle connecting the starting position with the final handle position (1000 ms after the target jump) as the movement direction. As we will demonstrate in Results, there was a linear relationship between the target and movement directions. Therefore, to represent how the participants corrected movement at the end of the trial, we calculated the slope of the regression line for each participant as an index. This slope was compared between period (before and after distortion) and group (inward and outward adaptation) by a two-way repeated-measure ANOVA (statistical significance was delineated at *p* < 0.05 throughout the study).

To examine the rapid component of online movement correction, we analyzed the lateral component (i.e., *x*-component) of force exerted on the handle 170–200 ms after the target jump. The force output depended on the amount of target jump. Accordingly, we calculated the force output for each target jump (small, ±7.5°; medium, ±15°; large, ±30°). The force output for each target jump size was obtained by multiplying the slope of the regression line by the size of target jump. The calculated force output was compared between target jump sizes (small, medium, large), groups (inward and outward adaptation) and periods (before and after distortion) by a three-way repeated-measures ANOVA.

We also considered the influence of other factors, besides the change in the visuomotor map, on the feedback response. Specifically, we examined for changes in the kinematics of the movement to the central target that were caused by our visuomotor map distortion intervention, as even subtle changes in the kinematics could influence the feedback response (Franklin et al., 2012). To this end, we evaluated the peak velocity of the handle, as well as the lateral deviation of the handle at the peak velocity, to examine movement toward the central target when the cursor was visible (30 trials). Second, we evaluated whether the intervention caused changes in the cursor’s deviation from the targets. Greater deviations of the cursor from the targets would give the participants more opportunities to correct the movements online, which could strengthen the feedback response (Franklin and Wolpert, 2008). To evaluate this, we measured the lateral deviation of the cursor from a straight line connecting the start and target positions. This was performed for the trials in which the cursor was visible (100 trials each for the left and right targets, and 30 trials for the central target), and then the root mean-squared value was calculated for each participant. These values were compared between groups and periods using a two-way repeated-measures ANOVA.

For the data analysis of Experiment 3 investigating the offline movement correction, we calculated the aftereffect in the trial after the perturbation (i.e., visual rotation) trial. According to the force output data for the online movement correction in Experiment 2 (see Results), the online correction should start ∼130 ms after movement onset. To remove the influence of the online movement correction, the aftereffect was defined as the movement direction 120 ms after movement onset. The calculated aftereffect was compared between periods (before and after distortion) and groups (inward and outward adaptation) using a two-way repeated-measures ANOVA. We also compared movement error experiences for the trial in which the visual rotation was applied in order to confirm participants experienced the same amount of movement error.

We instructed participants not to correct their movement when the visual rotation was imposed; however, it is possible for online corrections to be induced implicitly (Goodale et al., 1986; Desmurget et al., 1999; Day and Lyon, 2000). Because online movement corrections are capable of influencing the offline movement corrections made in the subsequent probe trial (Kawato and Gomi, 1992), we evaluated how the online corrections for the visual rotation trials were different between groups (inward and outward groups) and periods (before and after distortion). To this end, as in Experiment 2, we analyzed the cursor’s direction at 500 ms after movement onset from the central target direction and the force output for the movement correction averaged from 170–200 ms after movement onset. A two-way repeated-measures ANOVA was used to compare these measurements across periods and groups.

## Results

### Artificial distortion of the visuomotor map

**Figure 3.**
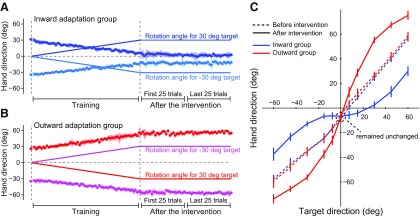
Distortion of visuomotor map. ***A***, ***B***, The imposed visual rotation (solid lines) and the movement direction of the hand in the trial for inward (***A***) and outward adaptation groups (***B***). The hand movements became closer (***A***) and more distant (***B***) to compensate for gradually increasing visual rotation. Data are represented by mean ± SD. ***C***, Visuomotor map before (broken lines) and after distortion (solid lines). The shape of the visuomotor map in the vicinity of the central target (0°) was distorted according to the training that each group received. Notably, movement toward the central target remained unchanged. Error bars represent SD.

In Experiment 1, we characterized in what way the visuomotor map was distorted before and after the respective interventions (inward and outward adaptation groups), before conducting Experiments 2 and 3. Figure 3*A* illustrates the trial-dependent change in the movement direction of the handle for the inward adaptation group (*N* = 6; rotations shown in Fig. 2*A*). Before visual rotation was imposed, the direction of movement was toward the respective target (either 30° to the right or left of the straight-ahead position). During the training period, participants in this group experienced an outward visual rotation, and compensated by directing their hand movement in the direction opposite to the rotation (inward). Movement direction became closer to the straight-ahead position as adaptation occurred (Hirashima and Nozaki, 2012) link to reference. Notably, participants were not aware of the visual rotations, as rotations increased gradually by 0.5° per trial. Figure 3*B* shows the trial-dependent change in the movement direction of the handle for the outward adaptation group (*N* = 6), which received visual rotations in the direction opposite that of the inward adaptation group (a leftward rotation occurred in trials involving the right target and a rightward rotation occurred in trials with the left target; Fig. 2*B*). In contrast with the inward adaptation group, participants corrected by directing cursor movement farther away from the straight-ahead position with increasing trials.

Before and after intervention, we measured the visuomotor map by asking participants to reach toward targets located at various positions (0, ±7.5°, ±15°, ±30°, ±45°, ±60°) without visual feedback from the cursor (Fig. 2*C*). As illustrated in Figure 3*C*, prior to intervention, movement directions were almost identical to target directions (broken lines). However, the shape of the visuomotor map was distorted depending on the type of intervention (Fig. 3*C*). For the inward adaptation group, the line corresponding to the visuomotor map became flatter around the central target (0°, solid blue line), implying a lowered sensitivity to changes in the target’s direction. Conversely, the line corresponding to the visuomotor map of the outward adaptation group became steeper, compared with before the intervention (solid red line), implying that sensitivity increased. Importantly, movement direction toward the central (0°) target remained unchanged by both interventions (Fig. 3*C*), indicating that it was unaffected by distortion of the visuomotor map. Therefore, this paradigm was suitable for use of the movement to the central target as a probe trial to compare online (Experiment 2) and offline (Experiment 3) movement corrections, before and after distortion of the visuomotor map.

### Gain alteration of online correction

**Figure 4.**
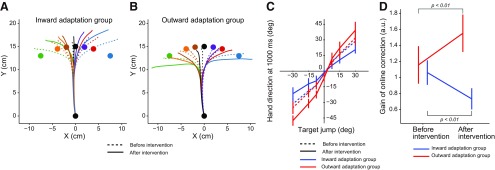
Online movement correction. ***A***, ***B***, Trajectories of the hand before (broken lines) and after (solid lines) distortion of the visuomotor map for the inward adaptation group (***A***) and outward adaptation group (***B***). Each color represents the trajectory for a discrete target jump. ***C***, Online movement correction was evaluated 1000 ms after the onset of target jump before and after distortion of the visuomotor map. ***D***, The slope of the linear relationship between size of the target jump and corrected movement direction (***C***) significantly decreased after distortion in the inward adaptation group, whereas it significantly increased in the outward adaptation group. Error bars indicate SD.

Experiment 2 was designed to investigate how online movement corrections were influenced by distortion of the visuomotor map. To this end, we randomly interleaved probe trials in which the central target suddenly jumped to peripheral locations (±7.5°, ±15°, and ±30°; Fig. 2*D*) immediately after movement onset. We compared the online movement corrections induced by the target jump between trials before and after distortion of the visuomotor map (Fig. 4*A*,*B*).

The degree of correction seemed to decrease or increase depending on the type of distortion each group received. In order to quantify the amount of online movement correction and ensure if the participants tried to correct the movement properly even when the visual information of the cursor was absent, we first determined the angle of the handle position relative to the starting position 1000 ms after the target jump (i.e., final position). Figure 4*C* indicates the relationship between target jump angles and resultant final movement angles. There was a linear relationship between the angles, from which we calculated the slope of the regression line as an index to quantify the degree of online movement correction. A two-way repeated-measures ANOVA revealed that there were significant interactions between period (before and after distortion of visuomotor map) and group (inward and outward adaptation; *F*_(1,14)_ = 94.06, *p* = 1.36 × 10^−7^; Fig. 4*D*). The simple main effect of period (before and after visuomotor map distortion) was also significant for both the inward (*F*_(1,14)_ = 38.76, *p* = 2.21 × 10^−5^) and outward (*F*_(1,14)_ = 56.10, *p* = 2.92 × 10^−6^) adaptation groups. Specifically, distortion of the visuomotor map resulted in diminished online corrections in the inward adaptation group and exaggerated online corrections in the outward adaptation groups (Fig. 4*D*).

**Figure 5.**
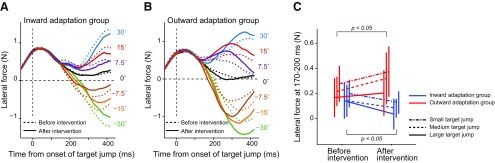
Rapid component of online movement correction. ***A***, ***B***, The *x*-component of force output exerted on the handle before (broken lines) and after (solid lines) distortion of the visuomotor map for the inward (***A***) and outward (***B***) adaptation group. The values shown on the right side indicate the size of target jump. ***C***, Force output was averaged between 170 and 200 ms after target jump for each size jump (small, dashed-dotted lines; medium, broke lines; large, solid lines). Error bars indicate SD.

Next, to see how early movement correction started, we examined the lateral force exerted on the handle during the online movement correction. Figure 5A and B indicates how the lateral force output changed with time. The force output for the movement correction appeared to emerge approximately 130 ms after the target jump. We averaged the force output between 170–200 ms after the target jump to examine how distortion of the visuomotor map influenced the force output for rapid online movement correction and grouped the responses for each of three target jump sizes (small, ±7.5°: medium, ±15°: large, ±30°). Analysis with a three-way repeated-measures ANOVA (group × target jump size × period) revealed no second-order interaction (*F*_(2,28)_ = 0.15, *p* = 0.859); however, there was a first-order interaction between period and group (*F*_(1,14)_ = 9.86, *p* = 7.24 × 10^−3^), which indicates that the two types of visuomotor map distortions differentially altered the force output for a movement correction. Indeed, there was a simple main effect of period in both the inward (*F*_(1,14)_ = 4.90, *p* = 0.044) and outward adaptation groups (*F*_(1,14)_ = 4.95, *p* = 0.043; Fig. 5*C*). The statistical results were not substantially different when the data for time windows after 170 ms (e.g., 175–205 ms, 180–210 ms) were analyzed. Thus, the rapid component of lateral force for online movement correction was decreased (inward adaptation group) and increased (outward adaptation group) depending on the visuomotor map distortion that each group received. Interestingly, as shown in Figure 5*C*, the corrected force outputs during this time window appeared to increase as the size of the target jump became smaller. This is in contrast with a recent study by Franklin et al. (2016), showing that the corrected force was correlated with the size of the target jump. However, the size of the target jump in their study was ∼7° at maximal, which was much smaller than the target jump in the present study. Thus, it is possible that the online correction was nonlinearly modulated by the size of the target jump. Indeed, Haith et al. (2015) reported that reaction time to a target jump was shorter for a 45° target jump than for target jumps at 90° and 135°. A similar nonlinear modulation has also been reported in the aftereffect, observed in the trial after the cursor is displaced (Wei and Körding, 2009; Kasuga et al., 2013).

**Figure 6.**
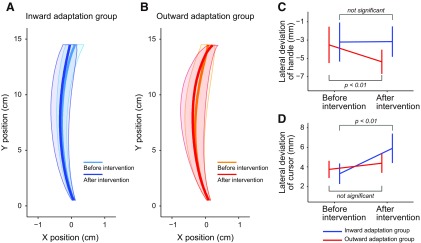
Kinematics of the movement. ***A***, ***B***, The trajectories of the handle during movement to the central target (the cursor was visible) before and after intervention for the inward (***A***) and for outward (***B***) adaptation groups are shown. Solid bold lines indicate the averaged trajectory of participants. Shaded areas indicate SD. Note that the scale for the *x*-direction is exaggerated. ***C***, The handle’s *x*-position during peak velocity was quantified to examine the shape of the trajectories. After intervention, the values became significantly smaller by 1.9 mm only for the outward adaptation group, indicating that the trajectories were slightly curved leftward. ***D***, The lateral deviation of the cursor’s direction at peak velocity from the target direction (left, central, and right targets) was quantified by taking the root mean squared value for each participant. After intervention, the deviation significantly increased only in the inward adaptation group. In ***C*** and ***D***, error bards indicate SD.

We examined whether changes in the online movement correction could be explained by factors other than distortion of the visuomotor map itself. A two-way repeated-measure ANOVA applied to the peak velocity of the handle toward the central target indicated that there was no significant main effect for group (*F*_(1,14)_ = 0.58, *p* = 0.46) or period (*F*_(1,14)_ = 0.15, *p* = 0.70), and no significant interaction between them (*F*_(1,14)_ = 0.26, *p* = 0.62). The movement trajectories toward the central target when the visual cursor was available are shown in Figure 6A and B. Although the trajectories before and after visuomotor map distortion largely overlapped (Fig. 6*A*,*B*), a two-way repeated-measures ANOVA, for deviations in the path of the lateral hand at the peak velocity, revealed a significant interaction between period (before and after the distortion of the visuomotor map) and group (inward and outward adaptation; *F*_(1,14)_ = 8.251, *p* = 0.012). There was a significant simple main effect of period only for the outward group (*F*_(1,14)_ = 15.64, *p* = 1.43 × 10^−3^; Fig. 6*C*). However, the difference was small (∼0.2 cm) and this effect was not observed in the inward group (*F*_(1,14)_ = 0.012, *p* = 0.92).

We also examined whether cursor movement deviated from the straight path to the target more or less in different groups and periods by calculating the root mean squared values of the lateral deviation for each participant. A repeated-measures two-way ANOVA revealed that there was a significant interaction between group and period (*F*_(1,14)_ = 10.322, *p* = 6.26 × 10^−3^), and there was a simple main effect of period for the inward adaptation group (*F*_(1,14)_ = 36.07, *p* = 3.22 × 10^−5^; Fig. 6*D*). This indicates that movement accuracy deteriorated in the inward adaptation group; however, there was no simple main effect of period for the outward adaptation group (*F*_(1,14)_ = 2.139, *p* = 0.17; Fig. 6*D*). Together, the procedure to distort the visuomotor map did not change the kinematics and/or the movement accuracy sufficiently to explain the changes observed in the online movement correction.

### Gain alteration of offline correction

**Figure 7.**
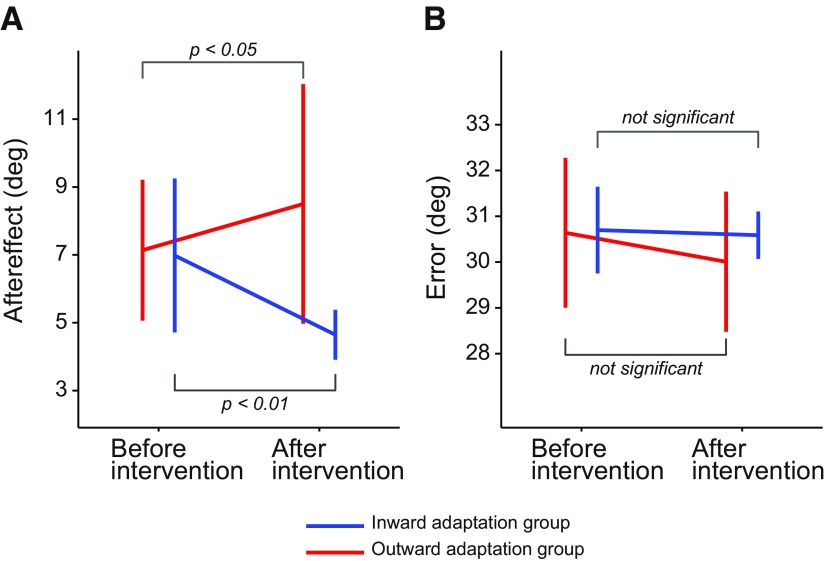
Offline movement correction. ***A***, The aftereffect was quantified in the trial immediately after the perturbation trial (it was evaluated as the movement direction 120 ms after movement onset). The aftereffect significantly decreased after distortion of the visuomotor map in the inward adaptation group, whereas it significantly increased in the outward adaptation group. ***B***, Visual errors in the perturbation trial (the errors should be 30º) were not significantly different before and after intervention. Error bars indicate SD.

Experiment 3 was designed to examine how distortion of the visuomotor map influenced offline movement correction. To this end, after either intervention, we interleaved visually perturbed trials (i.e., the cursor was rotated by ±30° around the starting position) and examined the aftereffect observed in the next trial (Fig. 2*E*). Figure 7 shows the aftereffects for both groups and for both periods. A two-way repeated-measure ANOVA revealed that there was a significant interaction between group and period (*F*_(1,20)_ = 18.54, *p* = 3.43 × 10^−4^). Furthermore, there was a significant simple main effect of period in the inward (*F*_(1,20)_ = 14.80, *p* = 1.00 × 10^−3^) and outward adaptation group (*F*_(1,20)_ = 5.03, *p* = 0.037), indicating that the aftereffect was decreased (inward adaptation group) and increased (outward adaptation group) depending on the distortion of visuomotor map. A two-way repeated-measures ANOVA was also applied to the error that each group received in the visual rotation trials. There was no significant main effect for group (*F*_(1,20)_ = 0.48, *p* = 0.50) or period (*F*_(1,20)_ = 2.07, *p* = 0.17), and no significant interaction between the two (*F*_(1,20)_ = 1.02, *p* = 0.33), indicating the amount of sensory prediction error itself did not differ between groups and between periods. Again, the effects of group and period on offline movement correction were not explained by differences in the kinematics of the handle; a two-way repeated-measures ANOVA applied to the peak velocity of the handle toward the central target indicated that there was no significant main effect for group (*F*_(1,20)_ = 1.20, *p* = 0.29) or period (*F*_(1,20)_ = 2.42, *p* = 0.14) and no significant interaction between them (*F*_(1,20)_ = 1.72, *p* = 0.20).

**Figure 8.**
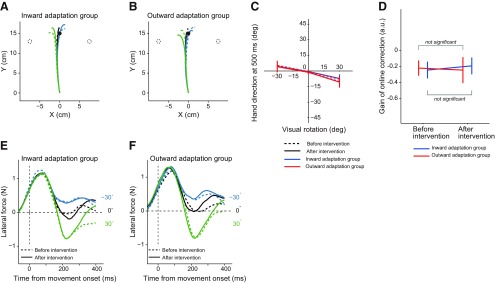
Online movement corrections in the perturbation trial in Experiment 3. ***A***, ***B***, The trajectory of the handle during movement to the central target in the perturbation trials, before and after the intervention, for the inward (***A***) and outward (***B***) adaptation groups is shown. The dotted circles indicate the corresponding final positions if movements were fully corrected. ***C***, Online movement correction was evaluated 500 ms after movement onset, before and after distortion of the visuomotor map. ***D***, The slope of the linear relationship between the size of the visual rotation and the corrected movement direction did not change by distorting the visuomotor map, for both experimental groups. Error bars indicate SD. ***E***, ***F***, The *x*-component of the force output exerted on the handle before (broken lines) and after (solid lines) distortion of the visuomotor map are shown for the inward (***E***) and outward (***F***) adaptation groups from movement onset.

We also examined how participants tried to correct their movement during the visual rotation trials. Analysis of the handle trajectories indicated that participants tried to minimize their movement corrections (Fig. 8*A*,*B*) in accordance with the instructions. An evaluation of movement direction, at the end of the movement, indicated that a slight correction was made (Fig. 8*C*,*D*). However, a two-way repeated-measures ANOVA showed that the degree of correction was not significantly different before and after the intervention (*F*_(1,20)_ = 0.413, *p* = 0.53 for the main effect of period; *F*_(1,20)_ = 3.404, *p* = 0.08 for the interaction between group and period). The force output for fast online corrections was also analyzed (Fig. 8*E*,*F*). No significant change in the average of the force output, from 170 to 200 ms after movement onset, was observed (*F*_(1,20)_ = 1.197, *p* = 0.287 for the main effect of period; *F*_(1,20)_ = 1.323, *p* = 0.264 for interactions between group and period by a two-way repeated-measures ANOVA). Thus, the procedure to distort the visuomotor map did not change how the participants corrected their movement during the visual rotation trials.

## Discussion

To consistently perform accurate reaching movements, the motor system has two kinds of mechanisms for movement correction within a trial (online correction) and in the next trial by motor adaptation (offline correction). Notably, these corrections could be achieved implicitly, as previously described (Goodale et al., 1986; Kagerer et al., 1997; Desmurget et al., 1999; Kasuga et al., 2013). We hypothesized that the visuomotor map is important for feedforward control during voluntary movement and plays a pivotal role in teaching the motor system how the movement should be corrected.

We distorted the shape of the visuomotor map by applying the opposite visual rotations to the cursor when reaching to two targets. After inward visual rotations were imposed when the participants aimed at two different targets located at ±30°, the actual movement direction became closer (Fig. 3*A*) and the visuomotor map around at 0° became flatter (Fig. 3*C*); the participant performed almost the same straight-ahead movement even when trying to reach peripheral targets (inward adaptation group). In contrast, the change in movement direction was more exaggerated after the participants experienced outward visual rotations (outward adaptation group; Fig. 3*B*,*C*). Importantly, these procedures did not change the reaching movement to the central target located at 0° (Fig. 3*C*). Thus, the changes in online and/or offline movement corrections for movement toward this target can be attributable to the distortion of the visuomotor map around 0°. It should be noted that all participants were unaware of the presence of visual rotation throughout the experiment, because the degree of visual rotation was increased so gradually. Adherence to this procedure is critical to distort the visuomotor map effectively; if visual rotation is imposed abruptly the participants might intentionally aim askew of the target position, as part of an explicit strategy (Taylor et al., 2014). In addition, if the participants happen to adapt to visual rotation by developing a strategy of moving the handle straight forward to the cursor located ±30°, then the adaptation does not necessarily result from an alteration of the visuomotor map, but rather from changing their aim.

Consistent with our hypothesis, we found that the degree of online and offline movement correction was altered according to the shape of the distorted visuomotor map. More specifically, in the inward (or outward) adaptation group, the degree of correction was reduced (or increased). These results indicate that the feedback controller and motor adaptation system refer to the visuomotor map that is used for feedforward control, suggesting a new perspective on the relation between feedforward control, feedback control, and motor adaptation.

### Factors influencing the feedback response

It is important to note that there are several other factors, besides the visuomotor map, that could potentially influence the feedback response. First, changes in the kinematics of the movement to the central target can induce different responses (Franklin et al., 2012). However, there were no statistically significant differences in the peak velocity of the handle during movement toward the central target, when the visual cursor was available, between groups (inward and outward groups) and periods (before and after interventions). Furthermore, these trajectories largely overlapped (Fig. 6*A*,*B*). The lateral deviation at the peak velocity was slightly (∼0.2 cm) different before and after the intervention in the outward adaptation group; however, this effect was not observed in the inward group (Fig. 6*C*). Therefore, differences in the kinematics of movement to the central target cannot fully explain the changes in the online movement correction caused by distortion of the visuomotor map.

Second, the procedure used to distort the visuomotor map could also change the movement accuracy. For example, if the movements by the outward adaptation group became more inaccurate after the intervention (i.e., the cursor deviated from the target path more often), the participants would need to correct their movement more often. Such increased opportunities to correct movement can change the rapid feedback response. Indeed, Franklin and Wolpert (2008) reported that the online response to a cursor jump was enhanced after repeated exposure to cases in which perturbed cursor movement needed to be corrected to reach a target, and could similarly be suppressed by cases in which the cursor was perturbed but did not need correction. However, as shown in Figure 6*D*, movement accuracy was maintained in the outward adaptation group, even after intervention. In contrast, accuracy was deteriorated by the intervention in the inward group (Fig. 6*D*), suggesting that these participants needed to correct their movement more often. Nevertheless, the online correction was decreased for the inward adaptation group (Figs. 4, 5), which opposes the prediction made by Franklin and Wolpert (2008). Together, the changes in the online correction observed in the present study are most likely caused by distortion of the visuomotor map, rather than indirectly by changes in the kinematics and/or an increased opportunity to correct the movement deviations.

### Relationship between feedforward and feedback control

Recent several studies have shown a close link between feedforward and feedback controls. Wagner and Smith (2008) demonstrated that after reaching movements were adapted to a velocity-dependent curl force field, the lateral force response to the suddenly imposed increase/decrease in hand movement also changed. In the visuomotor adaptation domain, Saijo and Gomi (2010) also reported the change in the feedback gain by adaptation to a visual rotation. These studies indicate that the feedback correction somehow reflected the acquired feedforward movement control. However, the muscle activity or movement direction of the hand for the probe trials differed before and after the motor adaptation, which makes interpretation of the feedback gain change before and after the adaptation difficult.

To overcome this possible criticism, Cluff and Scott (2013) developed a paradigm in which the kinematics and muscle activities during probe movement trials remained unchanged before and after the adaptation. They demonstrated that the long-latency reflex induced by perturbation to an arm, which might reflect the gain of feedback control Pruszynski and Scott, (2012), was enhanced after adaptation to a novel dynamic environment and concluded that the changes in the long-latency reflex truly resulted from motor adaptation.

The present study took a similar strategy; the kinematics of the probe trials remained unchanged before and after distortion of the visuomotor map. Consistent with previous studies showing the close link between feedforward and feedback controls, our results indicated the shape of the visuomotor map for feedforward control constrained the online movement correction (feedback control gain; Figs. 4, 5). Notably, we demonstrated that the feedback control gain could be enhanced or reduced according to the distortion of visuomotor map (Figs. 4, 5). The occurrence of both gain facilitation and reduction also indicates that changes in online movement correction were not merely due to habituation, sensitization, and/or fatigue effects caused by repetitive exposure to target jumps.

A recent prevailing optimal feedback control theory is a powerful scheme explaining many phenomena in voluntary movement control (Todorov and Jordan, 2002; Liu and Todorov, 2007; Izawa and Shadmehr, 2008; Nagengast et al., 2009). The optimal feedback control theory does not explicitly assume the separate presence of feedforward (inverse model) and feedback controllers. Rather, the controller is assumed to consist of a generic feedback controller with the help of the forward model and state estimator (Scott, 2004; Shadmehr and Krakauer, 2008). The results that the feedback and feedforward control were not completely separable are also consistent with this scheme.

### Offline movement corrections were not influenced by the alteration of online movement correction in the preceding trial

When we demonstrated that offline movement corrections were altered by the shape of the visuomotor map, we assumed that modifications of the offline movement correction occurred independently. However, if the online movement correction influenced the offline movement correction, as predicted by feedback-error learning (Kawato and Gomi, 1992), then alterations of the offline movement correction could be partly ascribed to the online movement correction in the preceding trial.

To exclude this possibility, we reduced the online feedback by setting the movement velocity in Experiment 3 to a faster speed than in Experiment 2. In addition, we instructed participants not to respond to the visual rotation of the cursor as this could suppress their response, although the earliest part of the responses remain unchanged by the instruction (Day and Lyon, 2000). In accordance with this, the online corrections were largely suppressed, as shown in the trajectories (Fig. 8*A–D*). Although the force outputs for fast online corrections were still present, we did not observe alterations in the online corrections that were caused by distortion of the visuomotor map (Fig. 8*E*,*F*). That distortion of the visuomotor map did not result in any modulations to the online corrections appear to be inconsistent with the results of Experiment 2. However, it is possible that instructing participants “not to respond”, and/or differences in the characteristics of perturbation (perturbation was suddenly applied or gradually increased for target jumps or visual rotation, respectively), made the modification ambiguous and suppressed their response. Regardless, the data clearly indicated that modulation of the online movement correction did not cause the modulation of the offline movement correction in the subsequent trial.

### Influence of shape of visuomotor map on motor adaptation

Recently, the degree of adaptation has been shown to be modulated by a wide variety of factors including prior experiences of perturbation (Braun et al., 2009; Huang et al., 2011; Kobak and Mehring, 2012), training schedules (Orban de Xivry and Lefèvre, 2015; Takiyama et al., 2015), type of visual feedback (Kasuga et al., 2013), delay of visual feedback (Tanaka et al., 2011; Honda et al., 2012), and reward and/or punishment information (Galea et al., 2015).

Structural learning is a recent influential idea (Braun et al., 2009; Kobak and Mehring, 2012) in which the motor system comprehends a perturbation structure by experiencing a randomly changing perturbations. This knowledge increases the adaptation speed when a constant perturbation is later imposed. In other words, the motor system learns to learn through experience (Braun et al., 2010). This notion is similar to our study, because our results indicate that the visuomotor map tells the motor system how the movements should be adapted. However, their scheme cannot explain our results, because both of our participant groups experienced the same visual rotations when they reached to the central target. Furthermore, the inward adaptation group demonstrated a reduction in aftereffect, implying that the adaptation speed could not necessarily be increased, but could be decreased. Our experimental results thus indicate a novel aspect of motor adaptation.

It would be also interesting to consider our results from the perspective of model-free learning; Huang et al. (2011) have reported that repeated successful movements to a particular direction, even after the effect was washed out, made subsequent visuomotor adaptation in this movement direction significantly faster. Thus, the repetitive movement direction worked as an attractor for adaptation. In our inward adaptation group, the forward movements could become an attractor, because this movement was repeated after the intervention. Thus, the movement to the central target could hardly escape from the forward movement, which might contribute to reducing the aftereffect. However, we interpreted that the decrement of aftereffect was likely to be caused by the distortion of visuomotor map rather than by the repetition of the movements. If the repetition of the movements was a main factor, it was hard to explain the result in the outward adaptation group that the aftereffect became greater after the intervention, because the movement to the central target was not repeated in this group. We assumed that the distortion of visuomotor map contributed to make the movement to the central target more escapable. However, future studies are necessary to provide mechanistic or theoretic explanations for how the repetition of movements could influence motor adaptation by examining the relationship between our scheme, structural learning, and model-free learning.

### Significance of establishing visuomotor map

In our scheme, the motor system cannot correct movement appropriately if a visuomotor map has not been established. Consider an example in which a player practices by swinging a baseball bat at a ball thrown in a constant place. Even if the player acquires skill, it does not directly indicate that she/he will do well in the real situation. Without establishing the appropriate map between the positions of ball and bat, the player cannot correct the trajectory of the swing when the ball unexpectedly moves to a different place (e.g., split-fingered fastball). Furthermore, even if the swing is deviated, it cannot be appropriately corrected in the next trial. To establish the appropriate map, the player might need to practice swinging a bat to a ball thrown to various places. This idea is similar to the schema theory proposed in the field of sports psychology (Schmidt, 1975). Recently, Wu et al. (2014) demonstrated that motor variability enhances the motor learning speed. Greater variability (or greater exploratory movement) should increase the opportunity of limbs to move toward various locations; it could be helpful to establish the visuomotor map in the vicinity of the movement.

Together, our results demonstrate that both online and offline movement corrections reflect the shape of the visuomotor map and suggest a close link between feedforward control, feedback control, and motor adaptation. Motor adaptation modifies the feedforward controller (i.e., visuomotor map), but our results indicate the influence of motor adaptation on visuomotor map is not unidirectional, because the shape of the visuomotor map also influenced motor adaptation. Such bidirectional interaction between the feedforward controller and motor adaptation reveals novel dynamic aspects of motor learning.

## Synthesis

The decision was a result of the Reviewing Editor Trevor Drew and the peer reviewers coming together and discussing their recommendations until a consensus was reached. A fact-based synthesis statement explaining their decision and outlining what is needed to prepare a revision is listed below. The following reviewer(s) agreed to reveal their identity: David Franklin

General comments

The authors have investigated the change in feedback commands and the relative updating of changes in feedforward commands after changing the visuomotor map. Subjects adapted to visuomotor rotations while reaching to two targets which either made the hands move closer together (while the visual movements remained similar) or moved further apart. The authors state that this warps the visuomotor mapping between the visual trajectories and the physical trajectories. The authors then perturbed the visual target in the middle of movements and examined the changes in the trajectories that subjects produced, showing that the early responses to the target jump were either reduced or enhanced appropriately according to the change in the visuomotor mapping. Finally the authors show that on subsequent trials the update from one trial to another after a visual error in the trajectory (due to the imposition of another visual rotation) is also appropriately modified according to the visuomotor map. Overall the authors show that both the feedback and feedforward changes in motor commands have been affected by the modulation imposed by the visuomotor rotations. Overall this paper is well designed and written and contributes an important understanding of the interaction between feedforward and feedback motor commands.

Major comments: Reviewer #1

The study includes a large sample size and control condition. However, the results do not appear to help reach the stated goals of the study. The study outcomes are that after adaptation to un-noticed visual distortion of cursor motion, subjects changed the gain of both online correction and showed aftereffects after the transformation was removed during catch trials. However, the coincidence of both adaptations does not indicate a causal relationship. Moreover, one can argue that both experiments test the same mechanism of online correction. During catch trials, an individual with adapted online correction can use it to adjust the ongoing movement for a proprioceptively sensed deviation from the desired motion, which would look like an aftereffect.

Major Comments: Reviewer #2

The author's main contribution is to examine the change in the feedback responses after the modification of the visuomotor map. The authors need to clarify in exactly what way this is distinct from the modification of the visuomotor responses due to task-relevant or task-irrelevant visuomotor maps as shown in Franklin and Wolpert (2008, Journal of Neuroscience) and Franklin, Franklin and Wolpert (2014, Journal of Neurophysiology). It is easy to consider these effects exactly the same - the inward rotations of the current study corresponding to a task-irrelevant environment where visual corrections are unnecessary and the outward rotations corresponding to a task-relevant environment with enhanced feedback responses. The current work may be due to different mechanisms, but the authors do not discuss these papers and contrast the responses. Can the authors clarify how their work cannot be explained by the task-relevance of the visual environment (or that this previous work did not modify the visuomotor mapping)?

The authors in the current paper claim that the movements to the central target in experiment 2 are unchanged by the visuomotor rotations. However, little comparison of these movements across the groups of performed. The conclusion would be stronger if the authors could clearly show that the full temporal pattern of kinematics for the central target were identical throughout the movement in these two groups - even after the target jumps were introduced. This is a concern as Franklin and colleagues (2014, Journal of Neurophysiology) showed small but consistent differences in the kinematics on unperturbed movements after the introduction of a visual distortion. The author's current line of reasoning is that the kinematics and muscle activity on this central movement were identical, therefore not contributing to the change in the feedback responses. Although there is some evidence that the online feedback due to visual motion is not influenced by changes in background muscle activity (Franklin et al., 2012, Journal of Neurophysiology), the comparison between the central target movements needs to be clearly demonstrated in the results and/or supported in the discussion.

I am concerned with the design of experiment 3. Perhaps the details are not completely outlined. On lines 205-206 you state that you asked subjects "to then aim at the center target as accurately as possible and not use explicit strategy to change the movement direction." Was visual feedback available on this trial? If so how does the visual response not change the motor response? At what point in the movement was the direction of the movement determined as plotted in Figure 6?

More critically you state that you asked subjects not to correct during the movement so that online correction did not influence the offline movement (line 207-208). However, much of the paper is investigating involuntary feedback responses. It has been shown that the motor responses to shifts in the visual motion of the cursor are produced involuntarily (as stated in the manuscript itself) throughout the movement. Please clarify exactly how you can be certain that the responses in the movement do not correspond to these feedback responses simply because you asked the subjects not to respond?

Minor issues:

Lines 40-41: I disagree with the statement that little is known about how and why feedback and feedforward control mechanisms are interrelated.

Lines 58-59 &amp; 69-72: The definition here of the visuomotor map is too broad. It appears to include coordinate frame transformations, multi-sensory integration, motor planning and motor execution. The definition needs to be more specific, in order to base a hypothesis on it.

Lines 64-65: The distortion applied here is purely visual. The statement that the whole visuomotor map as defined in the manuscript is distorted during the experiment does not appear to be true.

Lines 91-93: Are both these questions important and answered by the experiment? The authors appear to answer the question of why the implicit motor system knows the appropriate movement direction themselves in the same sentence, to reduce movement error.

Lines 94-95: This hypothesis is not testable.

Lines 98-99: It is not clear if this distortion was actually applied in the experiment, or it is just a thought experiment.

Lines 100-101: This appears to be an obvious prediction. The argument that adaptation during online correction is independent of the subsequent feed-forward adaptation needs to be developed here. Furthermore, the alternative prediction is not provided.

Liens 220-221: According to SI, positive rotation is CCW.

Line 224: One second after the target jump appears to be too long to measure online correction. What is the rational for this time period? It appears that movement already ends at this time. Thus, this does not appear to be a measure of a fast and early online correction, but a measure of the final outcome of all possible corrections, including feed forward secondary movements, during a whole movement.

Lines 250-251: The rational for the Experiment 1 does not appear to be well developed. Multiple previous studies have shown that visual transformations can be learned by people.

Lines 250-283: This section contains a lot of repetition from Methods. It appears to be a control condition, but that is not very clear.

Lines 304-306: This conclusion is not clear. In particular, it is not clear how the perturbation of the final handle position can become lesser or greater. Isn't it a controlled parameter?

Lines 321-322: It is not clear what this interaction means.

Lines 355-360: I do not follow this line of argumentation. I do not see a difference between the motor system "knowing" and knowing "implicitly" the direction of motion of the arm relative to the target.

All figures: SE is not a standard measure of variability. SD or CI are usually shown.

Fig. 5C: It is counterintuitive that largest forces were applied to correct for the smallest jumps in the outward adaptation group. This needs addressing.
